# New Medical School in Louisville

**Published:** 1848-02

**Authors:** 


### NEW MEDICAL SCHOOL IN LOUISVILLE.

Letters from Frankfort received within the last ten days, (it is now January 24th), announce that the Legislature of Kentucky, had before them an application for a charter for a second Medical School in this city. It was made, in person, by Dr. Joshua B. Flint who, in 1840, was requested by the Board of Managers of the Medical Institute, to relinquish the Chair of Surgery; a fact which we mention, as throwing light on the motives and origin of the new enterprize; in reference to which, as public medical journalists, it is both our privilege and duty, to express those views of its necessity and its influence on medical education in the West, and especially in Louisville, which we have deliberately formed.

We say, then, that an additional school in the central parts (at least) of our great valley, is neither needed nor demanded. As Louisville is an eligible place for *a* Medical School, if that established by the city ten years ago, had not grown, and become respectable; if its professors had been incompetent, and, therefore, did not attract pupils; if the Trustees had retained them in office, notwithstanding a manifest failure; and thus rendered the advantages of this central position nugatory, and no other remedy existed, it would not only be admissible, but praiseworthy, now to institute another school. But these conditions do not exist. The tabular view, presented in the preceding article, shows, that in ten years, the class has advanced from eighty, to four hundred and six; increasing at a ratio of forty per cent.; although, during the time, eight new schools, within the States from which it derives pupils, came into existence. In this unprecedented increase, through so considerable a period of time, we have an expression of the confidence of the profession, from which there should be no appeal- The Louisville school has, then, fulfilled the highest destiny, which the city and State could have proposed for it; and not disappointed the hopes and wishes of the students and physicians of the States, from which its classes have been made up. Now, what more could the people of Kentucky and of the West, generally, desire? We feel well assured, that *they* do not desire any thing more. But there might be a state of things, which would *seem* to justify and even demand the establishment of a second school in this city. It might happen (but we do not expect it) that more students would flock hither, than could be well received, and successfully taught in the present school; in which event, the decision, at *first* view, would be, that a second school should be set up. Even then, however, such a step might not be for the welfare of the medical profession, and the benefit of society; for the reason, that the anatomical and clinical resources, might not be adequate to the wants of a greater number of students than now attend; in which case, if attempts were made to retain more, the advantages to the whole would be diminished, and the standard of attainment lowered. When, however, the population of the city shall have advanced fifty or one hundred per cent., the means necessary to the growth and prosperity of a second school will be brought into existence, and then it ought to be organized. Till then, every effort should be directed to the firm and triumphant establishment of the first.

But suppose we admit, that, without reference to the population of the city and the means of instruction, a second school ought to be established as soon as the present attracts more pupils than it can teach, that state of things has not arrived. Some of the European schools, have several hundred more than the present Louisville class, and for many years the University of Pennsylvania has ranged between four and five hundred; yet the number has not, by the profession at large, been regarded as excessive. For the preceding two years, the classes in this city, have not reached three hundred and fifty. This year alone, has the number risen to four hundred; and, *should* no other school be established, it may not, (indeed under a scarcity of money certainly *would* not) again exceed, should it even reach that number:—the state of things, then, which, for argument we admitted, does not at present exist, and (referring solely to the public good), the projected school ought not, therefore, to be called into existence.

We may be asked, however, have not X, Y, and Z the same right to teach as A, B, and C? Undoubtedly they have. The right is natural and inalienable; but the right to grant degrees, that shall be passports to public confidence, is conferred by society, in its legislative capacity. Lawyers are admitted to practise, under a license from a court, appointed by the State government; and physicians are admitted to practise, under a diploma granted by Trustees, deriving their power from the same government. But wherefore, is that government charged with authority to grant charters, if it is to exercise no discretion? Most obviously, the whole system proceeds on the theory, that it *is* to exercise such discretion; otherwise a charter, being a matter of course, becomes an absurdity. And what is the theory of its discretion? Just as certainly, the public good. That is the end of all. However private and limited any corporation may be, it is presumed to have some bearing on the public weal; which is promoted by its existence, and by the existence of other corporations, of a like kind, the aggregate influence of which on society will be felt for good. If not felt at all, they would not be maintained by legislative authority; if felt for evil, it would be madness for the State to continue to charter them. Now what we have said, is sufficient to show, that in the exercise of a wise and foreseeing discretion, the Legislature ought not to authorize the establishment of another school in this city, at *this* time, for its means, as we have intimated, are sufficient for one only. Undivided, they may, by a proper administration, maintain it in usefulness and respectability:—divided, it would sink in both, faster than its competitor could rise. But, suppose that the rate of decline in one, and of growth in the other, should be precisely the same? Would the interest of medical education gain by the operation? Every judicious medical man, in the entire west and south, would answer—NO! but the very reverse; for two inferior schools could no more make up for one superior, than two negatives in human affairs, could make a positive—whatever they may do in algebra. To have able medical teachings, there must be able teachers; but to have these, (permanently) there must be large classes. Small classes can no more *secure* teachers of ability—than inferior teachers can draw numerous classes. Multiply schools then, beyond the demand and the means of instruction, and they become filled with inferior men, whereby the injury, at last, falls upon society. The public good is sacrificed, to the benefit of individuals.

Anxious as we have long been, to see, at least *some one*, of the many schools of our great valley, ascend to the same level with the highest of the older States, we cannot but view with deep concern, whatever threatens to throw back, the only one which has, as yet, approached that platform; and we cannot doubt, that every deep-thinking physician, who is jealous of the honor of his profession, will unite with us in this feeling. He must desire to see among us, at least a single institution, so well appointed and furnished, in means and men, as to render it unnecessary for our pupils to seek eastern schools, for the purpose of completing their education. At this time the University of Louisville, is ahead of all the rest; and if its means of instruction should not be reduced, seems likly to reach, and remain at that elevation, which all should desire to see somewhere exhibited in the West. We would say, then, to the people of Kentucky and of the city of Louisville, do not throw embarrassments in its way; but correct the errors in its administration and strengthen its weak places, if any exist; watch over its interests; and allow it to accquire the maturity and strength that will render it a permanent ornament of the State, and the pride of the medical profession of the West, before you undertake another.

We have heard it said, that competition is necessary to this distinction, and entirely concur in the opinion. But it must not be a competition which takes away the means of success, while it stimulates to effort. It need not to be domestic competition. The pupils reside over the West and South, which have, as we have already seen, twelve other schools, one of which is in Kentucky. Could greater competition be desired, by those who have the largest confidence in its power? Is not a school at St. Louis, or Memphis, or Lexington, or Cincinnati, a rival to that of Louisville; and do not these schools incite each other to effort, precisely as if they were all in Louisville. Suppose the whole of them were transplanted to this city, can any one believe, that the consequence would be the greater growth of its own University? Or that, in the aggregate, they they would continue to have the five or six hundred pupils, now in them? So far from this, it may be safely affirmed, that all medical teachings would soon be at an end; for the available means, when thus cut up, would be insufficient for any. But if those means are, now, only sufficient for one good institution, it follows that the removal of any one of them, to this city, would inflict the same injury, in a proportionate degree; while the establisment of a new one, by augmenting the general competition, would be still worse, than the transfer of one of them, as, we lately understood, was in the contemplation of its professors.

With these remarks we dismiss the subject. As public journalists we have looked, in all we have said, to the interests of the medical profession in the West and South; and if we are not mistaken, every view we have presented, is such as a journal published out of the State, and edited by persons having no connexion, either with Louisville or its University, might have taken. If so, we have discharged our duty as humble guardians of the profession, and having, thus placed our journal *rectus in curia*, we dismiss the subject.

